# Cultivar-Dependent Effects of Non-*Saccharomyces* Yeast Starter on the Oenological Properties of Wines Produced from Two Autochthonous Grape Cultivars in Southern Italy

**DOI:** 10.3390/foods11213373

**Published:** 2022-10-26

**Authors:** Vito Michele Paradiso, Luigi Sanarica, Ignazio Zara, Chiara Pisarra, Giuseppe Gambacorta, Giuseppe Natrella, Massimiliano Cardinale

**Affiliations:** 1 Laboratory of Agri-Food Microbiology and Food Technologies, Department of Biological and Environmental Sciences and Technologies, University of Salento, S.P. 6 Lecce-Monteroni, 73100 Lecce, Italy; 2 Enolife s.r.l., Viale delle Industrie, 74020 Montemesola, Italy; 3 I.I.S.S. Istituto di Istruzione Secondaria Superiore Basile Caramia–Gigante, Via Cisternino, 284, 70010 Locorotondo, Italy; 4 Department of Soil, Plant and Food Sciences, University of Bari, Via Amendola 165/a, 70126 Bari, Italy

**Keywords:** non-*Saccharomyces* yeast starter, *Lachancea thermotolerans*, global warming, autochthonous grape cultivars, volatile compounds, Bombino nero, Minutolo

## Abstract

Global warming poses a threat to winemaking worldwide, especially in dry–warm regions such as Southern Italy. Must fermentation with non-*Saccharomyces* yeast starter is a possible approach to limit the negative effects of climate change, leading to desirable effects such as an increase in total acidity and/or aroma improvement. The aim of this study was to evaluate the effects of the use of a non-*Saccharomyces* starter (*Lachancea thermotolerans*) on the chemical and sensory properties of wines obtained by the the fermentation of two autochthonous Apulian grape cultivars, namely Bombino nero and Minutolo, as compared to the traditional *Saccharomyces cerevisiae*-driven fermentation. Bombino and Minutolo wines fermented with either *Lachancea thermotolerans* or *Saccharomyces cerevisiae* were characterized for their oenological parameters, volatile profiles, and sensory properties. Both chemical and sensory properties were affected by the yeast starter. Inoculation of *L. thermotolerans* increased sensory complexity, with different floral and sweet-like attributes for both cultivars. Bombino nero, a neutral cultivar, showed a clear effect on wine composition, with both an increase in lactic acid and a change in the volatile profile. On the contrary, the impact of *L. thermotolerans* was partially masked in Minutolo due to the strong primary aroma background of this highly terpenic cultivar. In this work, we evidenced a notable cultivar × yeast interaction, showing how generalizations of the effects of non-*Saccharomyces* yeasts on vinification are difficult to achieve, as they show a cultivar-specific outcome.

## 1. Introduction

It is known that climate change impacts an increasing number of aspects of everyday life and affects more and more strategic sectors, including wine production [1]. Global warming is increasingly affecting the quality of both grapes and wine [2]. A warmer climate often determines undesired effects on both fruit growth and maturation, leading to wines with different physicochemical characteristics such as high alcohol content and low acidity, as well as a changed aromatic profile, often not accepted by the market [2,3].

The Apulia region is located in Southern Italy, between the Adriatic and Ionian seas, in the middle of the Mediterranean basin, and is characterized by a warm climate. Apulia is a region of great oenological relevance, with a vine-growing area of about 113,000 ha and a yearly wine production of around 10 million hectoliters. In Apulia, more than 50 cultivars are grown, each one with its own history and characteristics [4]. This makes Apulia one of the biggest wine-producing regions in Italy [5].

This study focused on two local cultivars (cv.): “Bombino nero”, commonly used in the production of rosé wines, and “Fiano Minutolo”, also called “Minutolo”, an aromatic cultivar similar to Muscat of Alexandria (not to be confused with “Fiano di Avellino”) [6,7,8,9,10].

In recent years, the increase in temperatures in the Mediterranean areas has been showing an effect in particular on white and rosé wines, which are losing the typical freshness and are becoming less acidic, with a higher alcohol content (due to the increase in the sugar accumulation of grapes) and with a definitely flatter and compromised aromatic bouquet [3]. The approaches adopted to face these negative effects and to maintain the production of local white and rosé wines in warm areas can vary, and they include the use of non-*Saccharomyces* yeasts [11,12].

In the past, non-*Saccharomyces* yeast species were completely unwanted in the industrial winemaking process because they were considered responsible for spoilage at both sensorial and chemical levels. In the last few decades, a new trend emerged, with the selection of non-*Saccharomyces* strains for oenological applications [13]. In fact, it has been shown that the combined fermentation of *Saccharomyces cerevisiae* and non-*Saccharomyces* yeasts can lead to improved freshness and aromaticity, features that can be easily compromised with the increase in temperatures and global climatic change [14]. In the last decade, many commercial non-*Saccharomyces* yeasts became available for winemaking, the majority of which is represented by three species: *Torulaspora delbrueckii*, *Lachancea thermotolerans*, and *Metschnikowia pulcherrima* [12]. Among these three species, *Lachancea thermotolerans,* previously known as *Kluyveromyces thermotolerans*, is known for its capacity to produce very complex and intense aromatic wines, together with the possibility of producing lactic acid, improving the acidity and the freshness of wines, and providing bio-control against spoilage microorganisms [15,16].

All these features indicate the fermentation by *L. thermotolerans* as a suitable tool to both enhance the quality and complexity of white and rosé wines in warm areas and increase product differentiation [13,15,17,18]. The aim of this study was to evaluate the effect of *L. thermotolerans* as a starter for white wine vinification of Minutolo and Bombino nero grapes and to compare these wines with those obtained by traditional *S. cerevisiae*-driven fermentation.

## 2. Materials and Methods

### 2.1. Materials

Grapes from *Vitis vinifera* L. cv. Bombino nero and Minutolo (2021 vintage) were harvested at commercial maturity (180 ± 5 g·L^−1^) in a vineyard located in Locorotondo (Bari, Apulia, Italy). The *Lachancea thermotolerans* strain KL5A (KLEY-ROSE) and the *Saccharomyces cerevisiae* strain LF13V were provided by Enolife s.r.l. (Montemesola, Apulia, Italy). Diammonium phosphate (DAP), yeast derivative (Aminoarom), and pectolytic enzymes (Lisopec) were provided by Enolife s.r.l. (Montemesola, Apulia, Italy).

### 2.2. Winemaking

**Figure 1:**
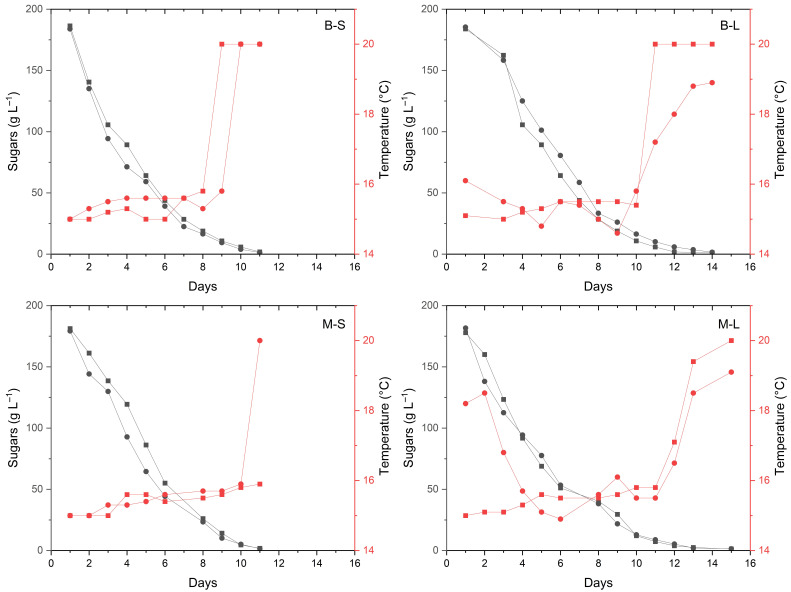
Fermentation curves of the wines obtained by the combination of grape cultivars (M—Minutolo, B—Bombino) and yeast starters (S—*Saccharomyces cerevisiae*, L—*Lachancea termotolerans*). Black lines and left *y*-axis: sugar consumption; red lines and right *y*-axis: temperatures. The two replicate fermentations per each combination of cultivar/starter are plotted independently and identified with circles and squares, respectively.

The following combinations of cultivar×yeast starter were tested: Bombino×*S. cerevisiae*, Minutolo×*S. cerevisiae*, Bombino×*L. thermotolerans*, Minutolo×*L. thermotolerans*. Eight vinifications (two replicates per combination) were carried out in the vinification plant of the “Cantina sperimentale–I.I.S.S. Basile Caramia-Gigante” in Locorotondo (Bari, Apulia, Italy). Grapes (at least 400 kg per trial) were transported to the winery in plastic boxes, immediately destemmed and crushed, and potassium metabisulfite (8 g·hL^−1^) was added. Dry ice was used to minimize oxidation effects. Crushed grapes were pressed using a pneumatic press, and skin maceration was carried out for 2 h before draining free-run and press-run juice. Pectolytic enzymes (3 g·hL^−1^) were added to the juice, and static clarification was carried out at 7 °C for 24 h. The chemical parameters of the clarified juices were as follows for Bombino and Minutolo, respectively: sugars, 185 and 181 g·L^−1^; pH, 3.31 and 3.19; total acidity, 5.71 and 6.32 g tartaric acid L^−1^; volatile acidity, 0.01 and 0.01 g acetic acid L^−1^. Diammonium phosphate (DAP, 30 g·hL^−1^) and yeast derivative (30 g·hL^−1^) were added to the clarified juice before inoculation of the respective yeast starter (1 × 10^10^ CFU g^−1^; 20 g·hL^−1^), which was rehydrated according to the manufacturer’s recommendations. Fermentation was carried out in a cold room under controlled temperature (16–18 °C, Figure 1), in 100 L stainless-steel fermentation tanks, with dry ice to minimize oxidation. 

Once the fermentation of sugars was complete, wines were stabilized with sulfite, bottled, and stored until analyses.

### 2.3. Chemical Analyses

Ethanol (% vol), pH, total acidity (g tartaric acid L^−1^), volatile acidity (g acetic acid L^−1^), malic acid (g·L^−1^), lactic acid (g·L^−1^), residual sugars (g·L^−1^), total dry extract (g·L^−1^), and net dry extract (g·L^−1^) in juices/wines were determined by infrared spectrometry with Fourier transformation (FTIR) with a WineScan FT 120 (FOSS, Hillerød, Denmark), which was calibrated with wines analyzed in accordance with official OIV guidelines [19].

### 2.4. Analysis of Volatile Compounds

Volatile compounds were extracted by solid-phase microextraction (SPME), according to Cardinale et al. [20] with some modifications. Samples were weighed (1 ± 0.05 g) into 20 mL vials containing 0.2 g/mL of NaCl (to increase ionic strength) and sealed with a silicone/PTFE septum and an aluminum cap. Semi-quantitation was performed by adding internal standard (2-octanol). A mother solution obtained from the pure standard (Sigma Aldrich, Milan, Italy), with a concentration of 820 mg·L^−1^, was diluted to reach a final concentration of 8.2 μg·L^−1^, then 10 μL of this final dilution was added to the sample. Samples were loaded into a Triplus RSH autosampler (ThermoFisher Scientific, Rodano, Italy). Before extraction, stabilization of the headspace in the vial was obtained by equilibration for 10 min at 50 °C. The extraction was carried out using a divinylbenzene/carboxen/polydimethylsiloxane (DVB/CAR/PDMS) 50/30 mm SPME fiber assembly (Supelco, Bellefonte, PA, USA) at 50 °C for 30 min. The fiber was desorbed at 220 °C for 2 min in the injection port of the gas chromatograph, operating in splitless mode. The GC-MS analyses were performed using a Trace1300 gas chromatograph equipped with an ISQ Series 3.2 SP1 mass spectrometer. The compounds were separated on a Thermo capillary column VF-WAX MS (60 m, 0.25 mm, 0.25 mm), under the following conditions: injection port temperature, 220 °C; oven temperatures, 40 °C for 0.5 min then 3 °C min^−1^ to 210 °C with a final isothermal for 2 min. The mass detector was set at the following conditions: detector voltage, 1700 V; source temperature, 250 °C; ionization energy, 70 eV; scan range, 33–150 amu. Tentative identification of the peaks was performed with the Xcalibur v2.0 software, and in particular Qual Browse, by matching their spectra with the reference mass spectra of the NIST library. Semi-quantitation of the compounds was performed by the internal standard method, and the amounts were expressed as mg of 2-octanol equivalents liter^−1^.

### 2.5. Sensory Analysis

A panel composed of 6 winemakers (4 males, 2 females; aged 27–43) was recruited for the sensory evaluation, which took place in two sessions, each one including four wine samples. Wines were evaluated in duplicate and presented in random order at 18 ± 2 °C in ISO-standard glasses coded with 3-digit random numbers. Wines were submitted for quality evaluation of odor and flavor/taste (odor genuineness, odor intensity, odor persistence, odor harmony, flavor genuineness, flavor intensity, flavor persistence, sweetness, alcohol, acidity, saltiness, in-mouth harmony, body) on a 10 cm unstructured scale. Genuineness of odor and flavor was defined as the absence of defects perceived by either orthonasal and retronasal perception, respectively [21]. Odor harmony was defined as the balance among orthonasal perceptions, while in-mouth harmony was defined as the balance between aroma and taste [22,23]. Then the samples were submitted to a check-all-that-apply (CATA) evaluation of sensory descriptors. Panelists individually rated each wine in an open-plan sensory facility and took a 1 min forced break between each wine, while having access to water and plain crackers as palate cleansers.

### 2.6. Statistical Analysis

Two-way analysis of variance (ANOVA) with interactions, one-way ANOVA, and Tukey’s post hoc test were carried out with Origin Pro 2022 (OriginLab, Northampton, MA, USA). Heatmap with cluster analysis was constructed with Origin Pro 2022 (OriginLab, Northampton, MA, USA) on standardized data of the volatile compounds. Clusterization was applied to both wines and volatile compounds (clusterization method: Ward; distance type: squared Euclidean).

Correspondence analysis (minimum term frequency = 3) and co-occurrence network analysis (minimum term frequency = 3, filter edges = Jaccard, top 40 edges) were carried out on the results of CATA sensory analysis using the KH coder software [http://khcoder.net/en/ (accessed on 23 October 2022)].

Principal component analysis (PCA) of the data from both volatile compound analysis and sensory attributes’ frequency was performed with Origin Pro 2022 (OriginLab, Northampton, MA, USA).

## 3. Results

### 3.1. Wine Chemical Profiles

The fermentation curves of the wines obtained by the combinations of grape cultivars and yeast starters are reported in Figure 1. The sugar concentration in the musts (mean ± standard deviation) was 185 ± 1 and 180 ± 2 g·L^−1^ for Bombino and Minutolo, respectively. Compared to *S. cerevisiae*, Bombino and Minutolo musts inoculated with *L. thermotolerans* required respectively three and four more days to complete the fermentation. This trend has been already reported and could be explained by the lower ethanol tolerance of *L. thermotolerans* with respect to *S. cerevisiae* [24,25].

Regarding their chemical profile, wines differed in some analytical parameters (Table 1). The slightly higher sugar content of the must from Minutolo produced wines with slightly higher alcohol levels as compared to Bombino wines. Minutolo wines presented higher levels of net dry extract, comparable with literature data [26,27,28]. Minutolo wines also presented both higher volatile acidity (although the levels were acceptable in all cases) and lower lactic acid concentration than Bombino wines. Volatile acidity and lactic acid were also affected by the yeast: in fact, the inoculation with *Lachancea* led to wines with higher levels of both volatile acidity and lactic acid. A cv × yeast interaction was also observed for both volatile acidity and lactic acid content: the increase induced by *Lachancea* was higher in Bombino as compared to Minutolo wines. Glyceropyruvic fermentation, leading to the formation of lactic acid, was therefore enhanced by *Lachancea*. Literature data report even higher amounts of lactic acid produced by this yeast, up to 12.0 g·L^−1^ [29]. However, high intra-specific variability has been reported for *L. thermotolerans* strains, due to geography, ecological niches, and domestication events [29,30]. Therefore, generalizations regarding the oenological impact of *L. thermotolerans* should be avoided and conclusions should be strain-related. Moreover, our findings point out how the interaction with cultivar can lead to different outcomes. To the best of our knowledge, there is little information about this kind of interaction of *L. thermotolerans* with the grape cultivar. Beckner Whitener et al. [31] evaluated its effect on the early stage of fermentation of Sauvignon blanc and Syrah musts, focusing on the volatile metabolite profile: they reported some common traits in both varieties, but also some cultivar-specific effects.

### 3.2. Wine Volatile Compounds

**Figure 2:**
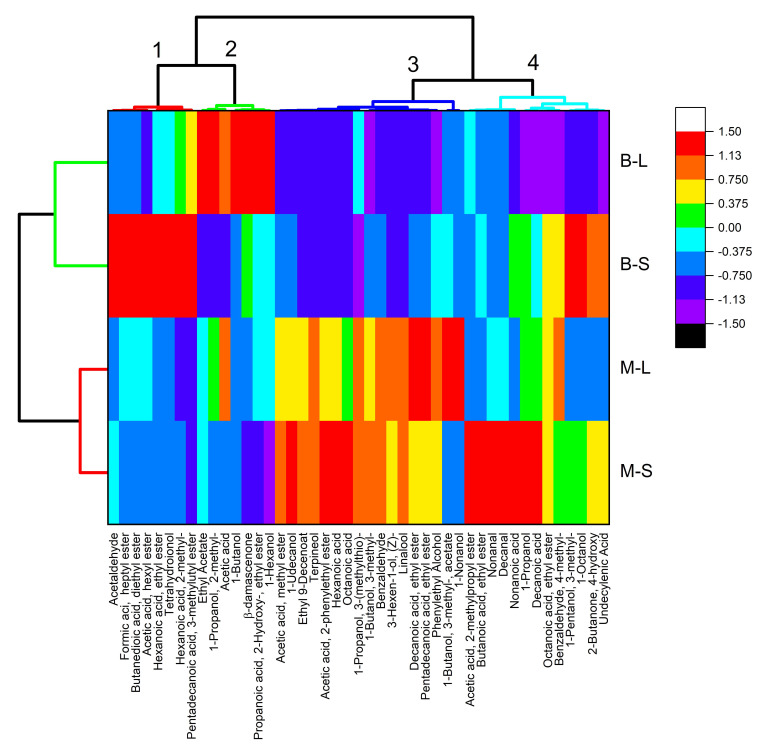
Heatmap and cluster analysis of the volatile compounds of the wines obtained by the combination of grape cultivars (M—Minutolo, B—Bombino) and yeast starters (S—*Saccharomyces cerevisiae*, L—*Lachancea thermotolerans*). The color map refers to the data of volatile compounds after standardization. Volatile compounds and wines are clusterized on the upper side and on the left-hand side of the heatmap, respectively. Clusters are indicated by different colors in the dendrogram. The numbers of the volatile clusters (1 to 4) refer to the discussion in the text.

The analysis of the volatile compounds evidenced an effect of both factors, the cultivar and the yeast starter, on the volatile profile of wines, as shown by the heatmap with cluster analysis (Figure 2). Heatmapping and cluster analysis showed different volatile profiles and dominant compounds between cultivars. The wines, in fact, were clustered according to the cultivar, indicating the prevalent impact of the cultivar and not the yeast used.

Volatile compounds were grouped into four different clusters. Considering the color scale of the heatmap, the clusters numbered 1 and 2 included volatile compounds found in higher levels in Bombino wines inoculated with *S. cerevisiae* and *L. thermotolerans*, respectively. The use of this non-*Saccharomyces* yeast strain, in particular, caused a shift in the volatile profile from esters (including esters with fruity notes such as ethyl-hexanoate and hexyl acetate [32]) to higher alcohols (including hexanol). Two different norisoprenoids, related to red fruit and violet notes, typical of red grape cultivars such as Bombino nero [33], characterized the two clusters. Inoculation with *L. thermotolerans* caused an increase in β-damascenone, a norisoprenoid considered an impact odorant for non-floral grapes [34], which has an aroma-enhancing activity [35,36] and was previously found at higher levels in wines obtained by co-fermentations with *L. thermotolerans* [37]. Bombino wines fermented with *S. cerevisiae* contained higher levels of tetrahydroionol, a norisoprenoid with violet notes [38].

Cluster number 3 included the volatiles mainly characterizing wines from Minutolo, irrespective of the yeast starter. This cluster included floral terpenes (linalool, terpineol), characteristic of the mildly aromatic variety Minutolo [27]; medium-chain fatty acids and their ethyl esters (related to fruity notes) [32,39]; isoamyl acetate, also associated with fruity notes [12]; and phenylethyl alcohol and its acetate, linked to flowery notes [37]. However, the use of the two different yeasts determined a shift of the prevalent volatile compounds, in particular of the esters. These findings are in agreement with previous research reporting reduced formation of fruity esters in the early fermentation volatile metabolite profile of both Syrah and Sauvignon blanc inoculated with *L. thermotolerans*, as compared to *S. cerevisiae* [31].

The last cluster included aldehydes, esters, acids and higher alcohols characterizing wines inoculated with *S. cerevisiae*, with a slight shift, evidenced by the color map (Figure 2), depending on the cultivar.

The substantially neutral character of Bombino allowed a clear differentiation of wines based on the inoculated yeast starter. On the contrary, the varietal impact of Minutolo on the volatile profile of the wines was greater than the impact of the yeasts.

Such interaction, together with the genetic variability among strains of the same species [29], explains the difficulty of finding unambiguous and generalized effects of non-*Saccharomyces* species in winemaking [13,40], while requiring specific evaluation of the combination cultivar × yeast strain [31]. In agreement with our findings, Hranilovic et al. [29] reported that the effect on the primary aroma compounds was particularly pronounced, as some of the most strain-affected volatile compounds (i.e., hexanol, octanol, β-damascenone) derived from grapes.

### 3.3. Sensory Analysis

**Figure 3:**
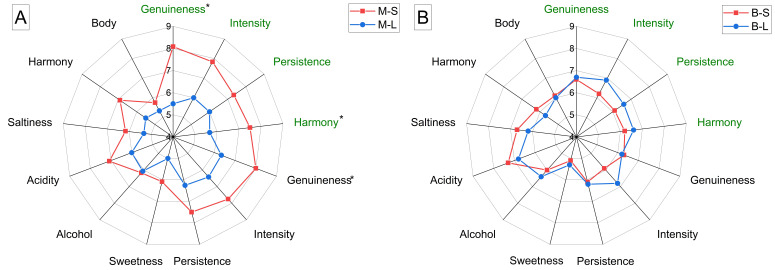
Radar graphs showing the results of the sensory evaluation of the wines produced from different combinations of grape cultivars (M—Minutolo, B—Bombino) and yeast starters (S—*Saccharomyces cerevisiae*, L—*Lachancea thermotolerans*). Minutolo (**A**) and Bombino (**B**) wines fermented with different yeasts. Odor descriptors are in green, taste/flavor descriptors are in black. For each parameter, significant differences between yeast starters are indicated with an asterisk (*p* < 0.05).

Figure 3 reports the judgments given to the wines by the panelists. Few significant differences were found when comparing wines of the same cultivar inoculated with different yeasts. Minutolo wines fermented with *L. thermotolerans*, in particular, obtained lower scores for olfactory and taste genuineness (defined as the absence of defects), as well as for olfactory harmony (Figure 3A). Panelists, therefore, perceived the impact deriving from the non-*Saccharomyces* yeast as not harmoniously integrated with the terpenic character imparted by Minutolo grapes to the wines. As regards Bombino wines, those produced with *L. thermotolerans* scored slightly higher for flavor intensity, persistence, and harmony than wines fermented with *S. cerevisiae*, though differences were not statistically significant.

**Figure 4:**
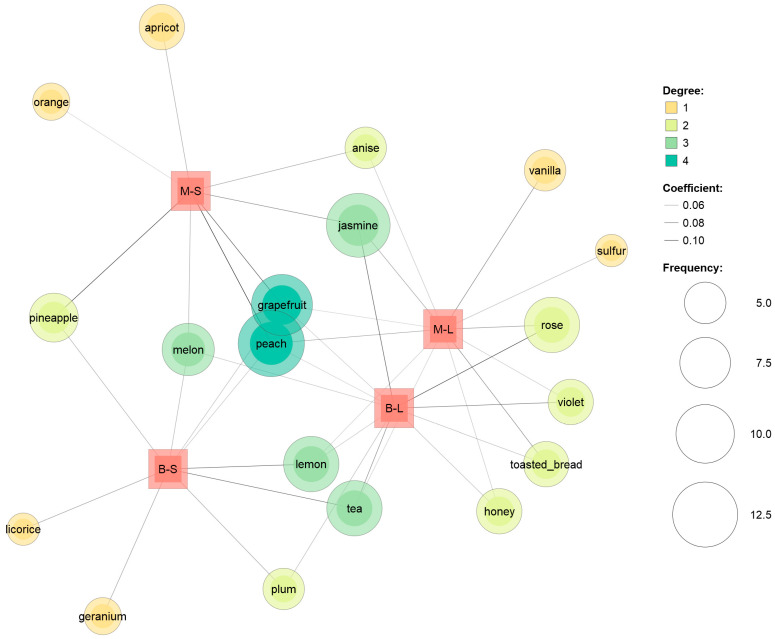
Co-occurrence network of the check-all-that-apply (CATA) attributes of the wines obtained by the combination of grape cultivars (M—Minutolo, B—Bombino) and yeast starters (S—*Saccharomyces cerevisiae*, L—*Lachancea thermotolerans*). The size of the circles represents the term frequency; the color of the circles represents the number of connected subjects (degree); the width of the connection lines is proportional to the Jaccard coefficient, indicating the strength of connections (see figure legend).

The results of the check-all-that-apply (CATA) analysis were subjected to co-occurrence network analysis (Figure 4). This analysis examined the potential relationships among wines represented with textual descriptors. To this end, this text-mining approach generated visual maps connecting subjects and descriptors on the basis of shared descriptors [41]. Only two attributes (peach and grapefruit) connected all four wines. Another frequent descriptor with floral notes (jasmine) connected Minutolo wines (characterized by varietal floral notes) and Bombino inoculated with *L. thermotolerans*. Lemon and tea notes connected Bombino wines and Minutolo inoculated with *L. thermotolerans*. The melon perception connected all wines except Bombino inoculated with *L. thermotolerans*. Bombino and Minutolo fermented with *S. cerevisiae* were mainly characterized by fruity notes and shared few attributes. Only one descriptor (pineapple) was exclusive to this couple of wines. On the contrary, wines fermented with *L. thermotolerans* shared four exclusive descriptors related to floral (violet, rose) and sweet-like/caramelized attributes (honey, toasted bread). The increased production of volatile compounds related to rose notes has been reported for *L. thermotolerans* [13,31]. Moreover, the different yeasts imparted exclusive attributes to Minutolo wines: either fruit and citrus (*S. cerevisiae*) or vanilla and sulfur (*L. thermotolerans*) notes.

**Figure 5:**
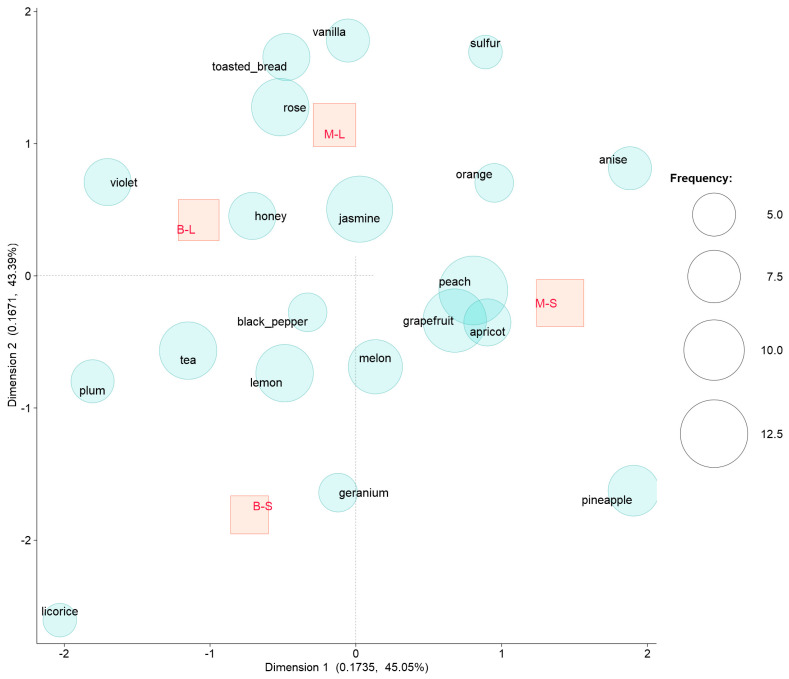
Correspondence analysis of the CATA attributes of the wines obtained by the combination of grape cultivars (M—Minutolo, B—Bombino) and yeast starters (S—*Saccharomyces cerevisiae*, L—*Lachancea thermotolerans*). Red squares correspond to wines, blue bubbles correspond to sensory attributes. Size of bubbles represents term frequency.

Correspondence analysis (CA) of the CATA textual descriptors, as reported by the panelists, showed a clear separation of the wines on the base of both cultivar and yeast factors (Figure 5).

Minutolo inoculated with *S. cerevisiae* presented fruity attributes (stone fruits, tropical fruits, and citrus), together with floral attributes, as a result of both varietal and fermentative contributions. The results are in agreement with the experimental data available on the sensory profile of Minutolo wines [42]. Bombino inoculated with *S. cerevisiae* showed less complexity, with black fruit (plum), herbaceous, and spicy attributes. This agrees with the neutral character of this red grape variety [33].

The use of *L. thermotolerans* starter led to a change in attribute patterns for both cultivars, adding both floral and sweet-like attributes. Bombino in particular showed increased aroma complexity, with both violet and honey attributes, while Minutolo was described by panelists with attributes of rose, vanilla, and toasted bread. Moreover, inoculation with *L. thermotolerans* caused an increase in the occurrence of the jasmine attribute in both cultivars. In a few cases, the panelists also reported a sulfur note.

**Figure 6:**
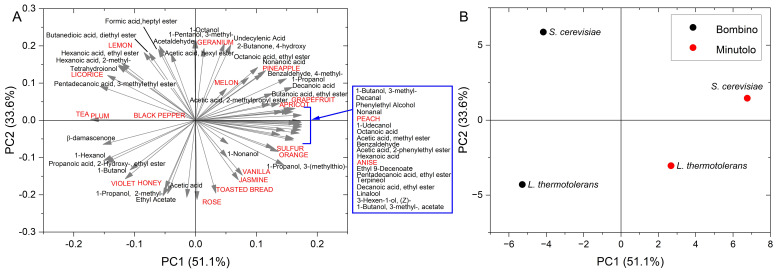
Principal component analysis ((**A**)—loading plot, (**B**)—score plot) of the volatile compounds (in black) and frequencies of sensory attributes (in red) of the wines obtained by the combination of grape cultivars (Minutolo and Bombino) and yeast starters (*Saccharomyces cerevisiae* and *Lachancea thermotolerans*).

The data from the analysis of volatile compounds were combined with the frequencies of sensory attributes obtained by the CATA analysis and submitted for principal component analysis (PCA) (Figure 6). The first two principal components accounted for 87% of the variability. The first principal component (51.1% of the variability) was linked to cultivar. Wines from Minutolo showed a richer volatile profile, including higher alcohols, ethyl esters, acetates, medium-chain aldehydes and acids, and terpenes. This rich volatile profile was linked to a variety of fruity attributes, including tree fruit, tropical fruit, and citrus descriptors. Even a sulfur attribute was reported for Minutolo wines, correlated to the levels of 3-(methylthio)-1-propanol (methionol). On the other hand, the plum, spicy, and vegetable attributes of Bombino wines were correlated with a profile characterized by norisoprenoids and some esters, including ethyl-hexanoate, ethyl lactate (2-hydroxypropanoic acid ethyl ester), and diethyl succinate (2,3-butanedioic acid diethyl ester). The effect of the yeast starter was mainly on PC2 (33.6% of the variability), though a slight decrease in the scores on PC1 was also observed. As also observed in the heatmap (Figure 2), the impact of the yeast strain was more evident in Bombino wines. The inoculation with *L. thermotolerans* in Bombino wines shifted the pattern of norisoprenoids and increased the levels of both ethyl lactate and ethyl acetate. This effect is consistent with that reported in the literature for wines inoculated with *L. thermotolerans* and could be associated with changes in sensory complexity [24,40,43,44]. In particular, both sweet-like and floral attributes were less perceivable, with an increase in rose perception and a differentiation between Minutolo (vanilla, jasmine, toasted bread notes) and Bombino (violet, honey notes).

## 4. Conclusions

A commercial strain of *L. thermotolerans* was compared with *S. cerevisiae* and tested as a starter for wines from two Apulian autochthonous cultivars (Bombino nero and Minutolo), with the aim of evaluating the effects on wine oenological parameters, volatile profile, and sensory properties, as well as the potential for product differentiation.

A strong interaction of the yeast starter with the cultivar was observed, regarding both oenological indices and volatile compounds, and consequently sensory evaluation. Inoculation of *L. thermotolerans* determined an increase in sensory complexity, with different floral and sweet-like attributes for both cultivars. The use of *L. thermotolerans* on Bombino nero, a neutral cultivar, showed a clear effect on wine composition, with an increase in lactic acid and a change in the volatile pattern. Minutolo, a mildly terpenic variety, gave wines strongly characterized by the varietal fingerprint, overlapping and partially masking the effect of the yeast starter. The sensory characteristics of Minutolo wines obtained using *L. thermotolerans* starter indicated the need for further strategies to improve the effect of such non-*Saccharomyces* yeast in mildly terpenic cultivars. Further studies are needed in order to validate the application of the *L. thermotolerans* strain KL5A, including the use of a mixed culture of *S. cerevisiae*–*L. thermotolerans* as well as sequential inoculation.

## Figures and Tables

**Table 1:** Chemical analyses of the wines obtained by the combination of grape cultivars (Cv. M—Minutolo and B—Bombino) and yeast starters (S—*Saccharomyces cerevisiae*, L—*Lachancea thermotolerans*). For each parameter measured (n = 2), the values indicate mean ± standard deviation; different capital letters indicate significantly different means (one-way ANOVA, main effects, *p* < 0.05); different lowercase letters indicate significantly different means (two-way ANOVA, interactions, *p* < 0.05).

Parameter	Minutolo		Bombino		Cv.		Yeast	
	S	L	S	L	M	B	S	L
Ethanol (% vol)	11.57 ± 0.03 a	11.66 ± 0.08 a	11.18 ± 0.07 b	11.29 ± 0.06 b	A	B		
pH	3.21 ± 0.03	3.29 ± 0.13	3.37 ± 0.00	3.36 ± 0.11				
Total acidity (g tartaric acid L^−1^)	6.49 ± 0.41	5.94 ± 0.27	5.45 ± 0.18	5.98 ± 0.91				
Volatile acidity (g acetic acid L^−1^)	0.19 ± 0.00 b	0.23 ± 0.00 ab	0.06 ± 0.00 c	0.28 ± 0.03 a	A	B	B	A
Malic acid (g·L^−1^)	1.00 ± 0.14	1.00 ± 0.14	1.00 ± 0.00	0.80 ± 0.42				
Lactic acid (g·L^−1^)	0.30 ± 0.00 b	0.40 ± 0.00 b	0.40 ± 0.00 b	1.50 ± 0.14 a	B	A	B	A
Residual sugars (g·L^−1^)	0.95 ± 0.08	1.51 ± 0.42	0.97 ± 0.01	2.03 ± 1.89				
Total dry extract (g·L^−1^)	19.50 ± 0.16	20.54 ± 0.51	18.09 ± 0.12	20.09 ± 2.19				
Net dry extract (g·L^−1^)	18.56 ± 0.25	19.03 ± 0.93	17.12 ± 0.12	18.06 ± 0.30	A	B		

## Data Availability

The data presented in this study are available on request from the corresponding author.
